# Identification of key modules and hub genes associated with lung function in idiopathic pulmonary fibrosis

**DOI:** 10.7717/peerj.9848

**Published:** 2020-09-08

**Authors:** Yuechong Xia, Cheng Lei, Danhui Yang, Hong Luo

**Affiliations:** Department of Respiratory Medicine, The Second Xiangya Hospital, Central South University, Changsha, Hunan, China; Research Unit of Respiratory Disease, Central South University, Changsha, Hunan, China; Hunan Diagnosis and Treatment Center of Respiratory Disease, Changsha, Hunan, China

**Keywords:** Idiopathic pulmonary fibrosis, Lung function, Weighted gene co-expression network analysis, Hub genes, Differentially expressed genes

## Abstract

**Background:**

Idiopathic pulmonary fibrosis (IPF) is a chronic and progressive interstitial lung disease, characterized by a decline in lung function. To date, the pathophysiologic mechanisms associated with lung dysfunction remain unclear, and no effective therapy has been identified to improve lung function.

**Methods:**

In the present study, we used weighted gene co-expression network analysis (WGCNA) to identify key modules and hub genes associated with lung function in IPF. Three datasets, containing clinical information, were downloaded from Gene Expression Omnibus. WGCNA was performed on the GSE32537 dataset. Differentially expressed gene s (DEGs) between IPF patients and healthy controls were also identified to filter hub genes. The relationship between hub genes and lung function was then validated using the GSE47460 and GSE24206 datasets.

**Results:**

The red module, containing 267 genes, was positively correlated with the St. George’s Respiratory Questionnaire score (*r* = 0.37, *p* < 0.001) and negatively correlated with the percent predicted forced vital capacity (FVC% predicted) (*r* =  − 0.46, *p* < 0.001) and the percent predicted diffusion capacity of the lung for carbon monoxide (Dlco% predicted) (*r* =  − 0.42, *p* < 0.001). Gene Ontology and Kyoto Encyclopedia of Genes and Genomes enrichment analysis suggested that the genes in the red module were primarily involved in inflammation and immune pathways. Based on Module Membership and Gene Significance, 32 candidate hub genes were selected in the red module to construct a protein-protein interaction network . Based on the identified DEGs and the degree of connectivity in the network, we identified three hub genes, including interleukin 6 (*IL6*), suppressor of cytokine signaling-3 (*SOCS3*), and serpin family E member 1 (*SERPINE1*). In the GSE47460 dataset, Spearman correlation coefficients between Dlco% predicted and expression levels of *IL6*, *SERPINE1*, *SOCS3* were –0.32, –0.41, and –0.46, respectively. Spearman correlation coefficients between FVC% predicted and expression levels of *IL6*, *SERPINE1*, *SOCS3* were –0.29, –0.33, and –0.27, respectively. In the GSE24206 dataset, all three hub genes were upregulated in patients with advanced IPF.

**Conclusion:**

We identified three hub genes that negatively correlated with the lung function of IPF patients. Our results provide insights into the pathogenesis underlying the progressive disruption of lung function, and the identified hub genes may serve as biomarkers and potential therapeutictargets for the treatment of IPF patients.

## Introduction

Idiopathic pulmonary fibrosis (IPF) is a chronic and progressive interstitial lung disease of unknown etiology, characterized by fibrosis or structural deformations, honeycomb lung, plaque pulmonary parenchymal fibrosis, and fibroblast foci (Raghu et al., 2011; Richeldi, Collard & Jones, 2017). The median survival time after diagnosis is 2–3 years (Kim, Perlman & Tomic, 2015; Martinez et al., 2017; Richeldi, Collard & Jones, 2017). To date, only pirfenidone and nintedanib have been approved by the United States Food and Drug Administration to treat patients with IPF. However, these treatments merely slow the progression of IPF, without improving lung function (Costabel et al., 2019; Costabel et al., 2017; Richeldi et al., 2014; Richeldi et al., 2020). Studies have confirmed that age, genetics, environmental factors, maladaptive repair processes, and the immune system are involved in the etiology of IPF (Martinez et al., 2017; Meng et al., 2020; Richeldi, Collard & Jones, 2017). However, the pathophysiologic mechanisms that underly IPF are complex and remain incompletely understood (Lederer & Martinez, 2018; Richeldi, Collard & Jones, 2017).

Transcriptomics studies of patients with IPF have demonstrated that transcriptional changes are involved in the pathophysiologic mechanisms of these diseases (Yang et al., 2007; Zuo et al., 2002). Genes that are differentially expressed in different groups are almost always associated with a particular disease phenotype (Huang et al., 2015; Konishi et al., 2009; Todd et al., 2019). Yang and colleagues (2013) analyzed the transcriptional profiles of lung tissue, collected from IPF patients and non-diseased controls, and found that the elevated expression of cilium genes was associated with more extensive microscopic honeycombing. Boon et al. (2009) studied the lung expression profiles of six patients with relatively stable IPF and six patients with progressive IPF, and found that genes associated with cell proliferation, migration, and cell morphology were highly expressed in the progressive IPF group relative to the stable IPF group.

The Gene Expression Omnibus (GEO; http://www.ncbi.nlm.nih.gov/geo) is a public database that provides a large quantity of gene expression datasets. These datasets can be downloaded freely and reused, to reveal the molecular pathogenesis of diseases. In this study, to identify key modules and hub genes associated with lung function in IPF, we downloaded datasets containing information regarding the clinical characteristics of lung function from GEO and performed weighted gene co-expression network analysis (WGCNA) on one dataset. Our study provides insights into the pathogenesis of progressive lung function decline in IPF, and the identified hub genes may represent therapeutic targets for the treatment of IPF patients.

## Materials & Methods

### Microarray data

**Figure 1:**
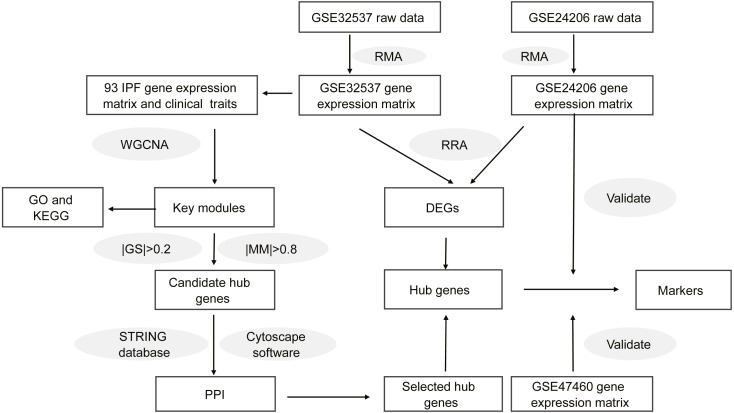
Workflow of this study.

Figure 1 shows the workflow of our study. On the GEO home page, “IPF” was used as the search term. We selected datasets according to the following criteria: (1) the gene expression profile was measured using microarray chip technology; (2) the samples for the study were lung tissues from healthy donors or patients with IPF; (3) the dataset provided raw data or a gene expression matrix; and (4) the dataset contained information regarding the clinical characteristics of lung function. Finally, we selected two datasets, GSE32537 and GSE47460. Although the GSE24206 dataset did not contain clinical characteristics, the patients with IPF were divided into two groups in this dataset. Lung samples that were obtained at the time of biopsy were considered to represent early IPF, whereas samples obtained at the time of explant were considered to represent advanced IPF (Meltzer et al., 2011). Therefore, the GSE24206 dataset was also included. Table S1 shows the details of the three datasets.

### Data preprocessing

We downloaded the raw data for GSE32537 and GSE24206. The raw data was obtained in CEL format. Data quality control was performed before data analysis. We used the affyPLM package (,http://www.bioconductor.org/packages/release/bioc/html/affyPLM.html, v.1.60.0) to assess the array quality, by calculating relative log expression (RLE) and normalized unscaled error (NUSE). We used the affy package (http://bioconductor.org/packages/release/bioc/html/affy.html, v.1.62.0) to normalize the array data by using the robust multi-array average (RMA) method. Then, we annotated the probes using platform annotation files, and genes that were represented by more than one probe were used to calculate the average gene expression levels. The gene expression matrix file for GSE47460, which had been normalized using a cyclic loess approach in pairwise fashion, was downloaded. We extracted IPF samples with complete data, including lung function [percent predicted forced vital capacity (FVC% predicted) and percent predicted diffusion capacity of the lung for carbon monoxide (Dlco% predicted)], and healthy donor samples from the GSE32537 and GSE47460 datasets. Table 1 shows the clinical characteristics of IPF patients in both datasets.

**Table 1:** Demographic data for subjects used in this study.

Characters	GSE32537 (93 IPF)	GSE47460 (86 IPF)
Age (years)	62.81 ± 8.32	63.65 ± 7.89
SGRQ score	46.81 ± 20.90	–
FEV1% predicted	–	68.20 ± 16.88
FVC% predicted	62.76 ± 15.86	61.38 ± 15.35
Dlco% predicted	46.76 ± 20.22	47.28 ± 18.71
Sex (%)		
Male	62(67)	61(71)
Female	31(33)	25(29)
Smoking history (%)		
Non-smoke	32(34)	30(35)
Former	61(66)	56(65)

**Notes.**

Data are presented as mean ± SD or *n*(%).

### Construction of co-expression network with WGCNA

Co-expression network analysis was performed using the R package “WGCNA” (https://cran.r-project.org/web/packages/WGCNA/index.html, v.1.69) (Langfelder & Horvath, 2008). First, we extracted the gene expression profile data from IPF patients in the GSE32537 dataset and selected the top 25% of variable genes. We constructed scale-free co-expression networks using these genes. Second, we used Pearson correlation matrices to calculate a correlation matrix among these genes. Third, we transformed the correlation matrix into a weighted adjacency matrix, through a power function. To construct scale-free networks, we chose the soft threshold power value, using the following criteria (Zhang & Horvath, 2005): (1) the generated Scale free Topology Model Fit R^2^ > 0.90; (2) the mean connectivity in the network should be as large as possible; and (3) the slope of the linear fitting model is around −1. Finally, we performed automatic network construction and module detection, using the following major parameters: power = 4, networkType = unsigned, maxBlockSize = 4,000, minModuleSize = 30, and mergeCutHeight = 0.25.

### Identification of clinically significant modules

Module eigengene represents the first principal component of a given module and the gene expression profiles in this module. When a sample trait is incorporated into the co-expression network, the correlation coefficient between the module eigengene and this sample trait can be calculated. The eigengene significance is defined as the correlation coefficient. Based on the eigengene significance, we were able to identify key modules.

### Gene ontology and KEGG enrichment analysis

To further understand the functions of the genes in the key module, Gene Ontology (GO) and Kyoto Encyclopedia of Genes and Genomes (KEGG) enrichment analyses were performed on the genes in the key module, using the R package “clusterProfiler” (http://www.bioconductor.org/packages/release/bioc/html/clusterProfiler.html,v.12.0) (Yu et al., 2012). We selected GO terms including biological process (BP), cellular component (CC), and molecular function (MF). We regarded a *p*-value <0.01 as the cut-off criterion.

### Identification of candidate hub genes in key modules

Module membership (MM) represents the intramodular connectivity of any gene in a given module. A higher absolute value of MM indicates that a gene has a higher negative or positive correlation with the module eigengenes (MEs). Gene significance (GS) is used to incorporate external information into the co-expression network. A higher absolute value of GS indicates the increased biological significance of a gene for a given clinical trait. Candidate hub genes in key modules were selected based on —MM—>0.8 and —GS—>0.2.

### Identification of DEGs

Differentially expressed genes (DEGs) between IPF lung tissues and healthy lung tissues were analyzed by the R package “limma” (http://www.bioconductor.org/packages/release/bioc/html/limma.html, v.3.42.2). DEGs were defined by —log _2_ Fold Change—>0.5 and adjusted *p*-value <0.05. During this process, the adjusted *p*-value, which is referred to as the false discovery rate (FDR), was calculated using the Benjamini–Hochberg correction method. Subsequently, the lists obtained from the differential expression analysis of each dataset were integrated, using the R package “RobustRankAggreg (RRA)” (https://cran.r-project.org/web/packages/RobustRankAggreg/index.html, v.1.1) (Bardou et al., 2014).

### Construction of a protein–protein interaction network

We uploaded candidate hub genes into the STRING database (https://string-db.org, v.11.0) to construct a protein-protein interaction (PPI) network, and we visualized the interaction relationships among the candidate hub genes using Cytoscape software (https://cytoscape.org, v.3.7.0). Finally, we identified hub genes according to the DEGs and the degree of connectivity within the network.

### Validation of hub genes

We validated the hub genes using the GSE47460 and GSE24206 datasets. The differential expression of hub genes between healthy lung tissue and IPF lung tissue in the GSE47460 dataset were calculated and visualized using the R package “ggpubr” (https://cran.r-project.org/web/packages/ggpubr/index.html, v.0.2.5). Then, we validated the correlations between lung function and expression levels of hub genes, using the R package “ggstatsplot” (https://cran.r-project.org/web/packages/ggstatsplot/index.html, v.0.3.1). We also validated the differential expression of hub genes between early IPF and advanced IPF using the GSE24206 dataset.

### Statistical analysis

Continuous variables were compared between two groups by applying the Student’s *t*-test or a non-parametric Wilcoxon rank-sum test, as appropriate. Associations between the expression levels of genes and lung function were determined by Spearman correlation coefficient. All statistical analyses were performed in R (v.3.6.1), and *p* < 0.05 was regarded as significant.

## Results

### Weighted gene co-expression network analysis (WGCNA)

**Figure 2:**
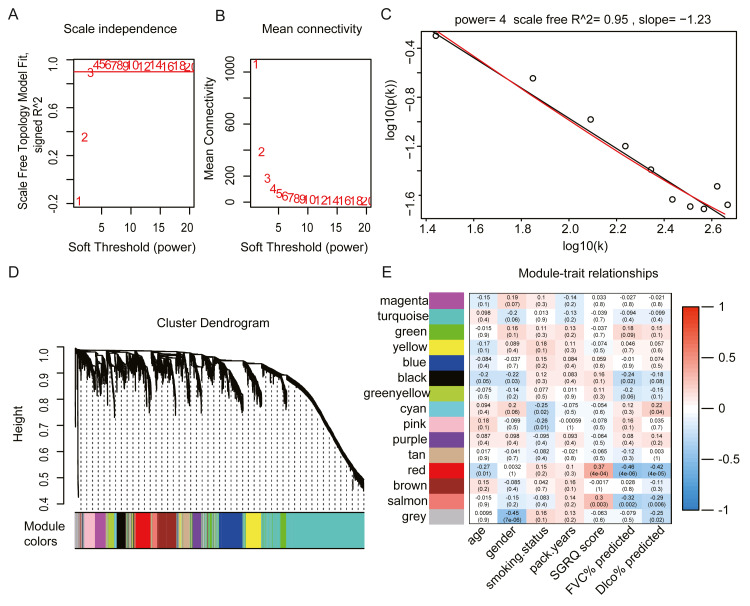
Construct gene co-expression network and identify key module in 93 IPF patients from GSE32537 dataset. (A–C) The process of selecting soft threshold. When we set *R*^2^ > 0.9, *β* = 4 is chosen, log-log plot of network connectivity distribution is almost a straight line, which represents that the network is approximately scale-free topology. (D) Cluster dendrogram and module color. branch represents gene cluster by average linkage hierarchical clustering and each color under cluster represents one co-expression gene module by the Dynamic Tree Cut. (E) Heatmap of correlation between module eigengenes and clinical traits. red color indicates positive correlation and green indicates negative correlation. In each cell, the up number represents correlation coefficients and the bottom number represents *P* value. The red module has the strongest negative correlation with lung function.

We selected the top 25% of variant genes identified in the GSE32537 dataset. A total of 93 IPF samples, containing 4,705 genes, were used for WGCNA. Hierarchical clustering analysis was performed, and when the threshold was set to 60, GSM806234 and GSM806335 were considered to be outliers (Fig. S1). Outlier samples were removed prior to further analyses. When the soft threshold power value was set to 4, the co-expression network exhibited an approximate scale-free topology (Figs. 2A–2C). WGCNA identified 14 modules, containing between 65 and 1,672 genes (Fig. 2D).

**Figure 3:**
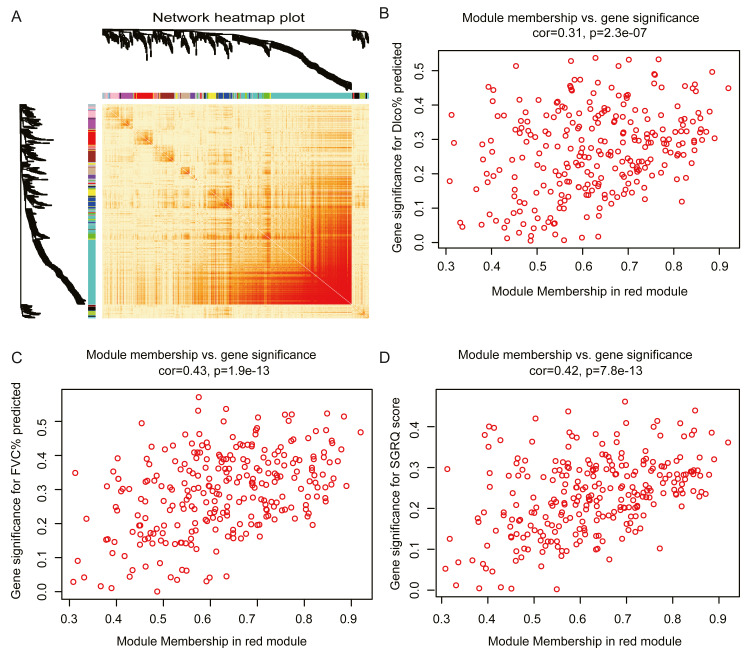
Module analysis. (A) Network heatmap plot of randomly selecting 400 genes. Branch represents gene cluster and each color under cluster represents one co-expression gene module. Light red indicates low overlap and darker red indicates higher overlap. The heatmap indicated the high independence between each module and genes at the tip of the module branches have high intramodular connectivity with the rest of the genes in this module. (B–D): scatter plot of gene significance (GS) for Dlco% predicted, FVC% predicted and SGRQ score versus module membership (MM) in the red module. GS is significantly correlated with MM, which indicated that the hub genes in the red module tended to be highly associated with lung function.

The red module had the strongest positive correlation with the St. George’s Respiratory Questionnaire (SGRQ) score (*r* = 0.37, *p* < 0.001) and was negatively correlated with FVC% predicted (*r* =  − 0.46, *p* < 0.001) and Dlco% predicted (*r* =  − 0.42, *p* < 0.001, Fig. 2E). The red module was identified as the key module. We randomly selected 400 genes, to visualize the relationship among the modules, using a heatmap plot showing topological overlap (Fig. 3A). The heatmap suggests a high degree of independence among the modules and genes at the tip of the module branches have high intramodular connectivity with the rest of the genes in this module. Then, the correlations between MM and GS for FVC% predicted, Dlco% predicted, and SGRQ score in the red module were shown in Figs. 3B–3D. GS showed a significant correlation with the MM in the red module, which indicated that the hub genes identified in the red module tended to be highly associated with lung function.

### GO and KEGG enrichment analysis

GO and KEGG pathway enrichment analyses were performed on the genes in the red module. GO enrichment results demonstrated that the red module genes were significantly associated with inflammation and immune responses, such as the response to lipopolysaccharide (LPS), leukocyte differentiation, cell chemotaxis, and the cellular response to molecules of bacterial origin. The KEGG pathway enrichment results indicated that genes in the red module were primarily enriched in the tumor necrosis factor (TNF) signaling pathway, the interleukin (IL)-17 signaling pathway, the Janus kinase (JAK)-signal transducer and activator of transcription (STAT) signaling pathway, and cytokine-cytokine receptor interactions. GO and KEGG terms were ranked in ascending order, based on *p*-values. Table 2 shows the top 10 categories associated with BP, CC, and MF. Table 3 shows the top 10 categories identified in the KEGG analysis.

**Table 2:** GO enrichment analysis in red module.

Category	ID	Description	*P*-value	Count
BP	GO:0002237	response to molecule of bacterial origin	4.95E−21	34
BP	GO:0032496	response to lipopolysaccharide	1.16E−20	33
BP	GO:0002521	leukocyte differentiation	8.93E−16	35
BP	GO:0060326	cell chemotaxis	1.29E−14	25
BP	GO:0050727	regulation of inflammatory response	2.41E−14	33
BP	GO:0048511	rhythmic process	2.32E−13	25
BP	GO:0071222	cellular response to lipopolysaccharide	4.61E−13	20
BP	GO:0071219	cellular response to molecule of bacterial origin	9.66E−13	20
BP	GO:0071216	cellular response to biotic stimulus	1.01E−12	21
BP	GO:0001819	positive regulation of cytokine production	4.26E−12	29
CC	GO:0101003	ficolin-1-rich granule membrane	1.39E−05	7
CC	GO:0070820	tertiary granule	5.88E−05	10
MF	GO:0001228	DNA-binding transcription activator activity, RNA polymerase II-specific	3.56E−11	26
MF	GO:0005125	cytokine activity	2.30E−07	13
MF	GO:0000978	RNA polymerase II proximal promoter sequence-specific DNA binding	2.61E−06	20
MF	GO:0070888	E-box binding	4.68E−06	7
MF	GO:0000987	proximal promoter sequence-specific DNA binding	5.15E−06	20
MF	GO:0030545	receptor regulator activity	7.72E−06	19
MF	GO:0050786	RAGE receptor binding	1.10E−05	4
MF	GO:0048018	receptor ligand activity	1.11E−05	18
MF	GO:0001227	DNA-binding transcription repressor activity, RNA polymerase II-specific	1.51E−05	13
MF	GO:0000980	RNA polymerase II distal enhancer sequence-specific DNA binding	2.74E−05	8

**Table 3:** KEGG enrichment analysis in red module.

ID	Description	*p*-value	Count
hsa04668	TNF signaling pathway	9.26E−16	21
hsa04657	IL-17 signaling pathway	1.11E−12	17
hsa04625	C-type lectin receptor signaling pathway	7.24E−10	15
hsa04380	Osteoclast differentiation	1.36E−08	15
hsa05144	Malaria	2.19E−08	10
hsa04060	Cytokine-cytokine receptor interaction	2.44E−08	22
hsa04933	AGE-RAGE signaling pathway in diabetic complications	3.17E−07	12
hsa04064	NF-kappa B signaling pathway	4.88E−07	12
hsa04630	JAK-STAT signaling pathway	1.89E−06	14
hsa04061	Viral protein interaction with cytokine and cytokine receptor	2.41E−06	11

### Candidate hub genes in the red module

**Figure 4:**
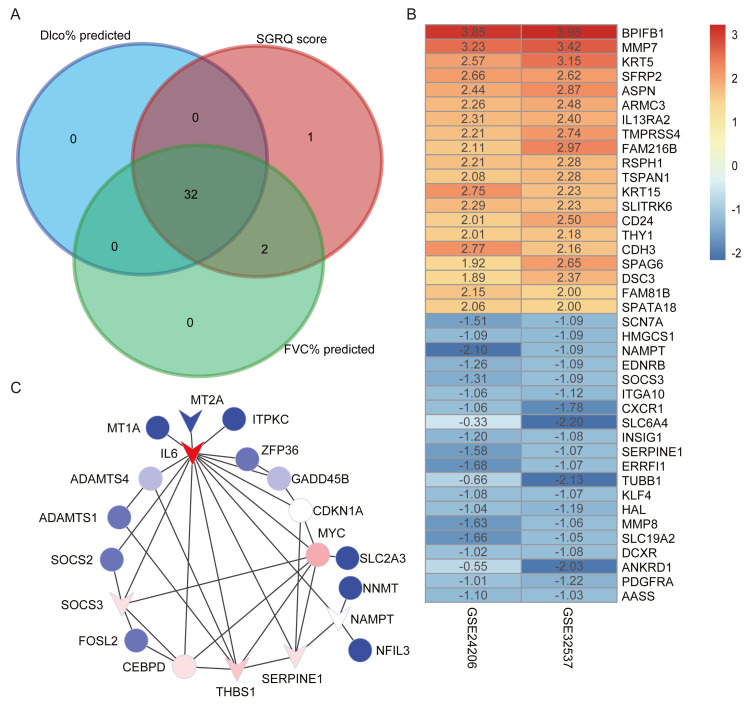
Construct PPI network and identify hub genes. (A) The intersection of candidate hub genes for clinical traits: Dlco% predicted, FVC% predicted and SGRQ score. (B) The top 20 upregulated genes and top 20 downregulated genes in IPF compared with healthy donors. The numbers in each rectangle show the logarithmic fold-change of genes in each dataset. Red represents upregulated gene and blue represents downregulated genes. (C) The PPI network visualized by Cytoscape software. We removed the 11 nodes which had no connections with others. The network contains 20 nodes and 35 edges. The triangle represented downregulation in IPF. Degree of connectivity was showed by different colors; the darker shade of red represents the higher degree of connectivity and, conversely, the darker shade of blue color indicates the lower degree of connectivity.

We selected candidate hub genes in the red module, based on the criterion: —MM— > 0.8 and —GS— > 0.2. Then we examined the intersection of the three gene lists (Fig. 4A). Finally, 32 genes were identified as candidate hub genes. Table 4 shows the GS and MM values for the 32 candidate hub genes in the red module.

**Table 4:** GS and MM of 32 candidate hub genes in red module.

	SGRQ score		FVC% predicted		Dlco% predicted			
Gene	GS	*P*-value	GS	*P*-value	GS	*P*-value	MM	*P*-value
NAMPT	0.36	4.34E−04	−0.47	2.90E−06	−0.45	8.02E−06	0.92	6.27E−38
GADD45B	0.32	1.98E−03	−0.31	2.75E−03	−0.30	3.47E−03	0.89	4.89E−32
THBS1	0.39	1.62E−04	−0.51	1.83E−07	−0.50	5.65E−07	0.88	3.46E−31
FOSL2	0.28	6.70E−03	−0.42	3.77E−05	−0.38	1.96E−04	0.88	2.49E−30
MYC	0.24	2.47E−02	−0.38	1.79E−04	−0.32	1.85E−03	0.87	2.99E−29
ITPKC	0.24	2.17E−02	−0.33	1.28E−03	−0.30	4.06E−03	0.86	5.16E−28
MT2A	0.28	7.17E−03	−0.44	1.55E−05	−0.33	1.41E−03	0.86	1.12E−27
NNMT	0.32	1.70E−03	−0.44	1.07E−05	−0.36	4.28E−04	0.86	1.59E−27
IL6	0.23	2.90E−02	−0.31	2.38E−03	−0.32	1.97E−03	0.86	1.96E−27
ERRFI1	0.29	4.82E−03	−0.38	2.04E−04	−0.38	2.27E−04	0.85	5.48E−27
SERPINE1	0.44	1.31E−05	−0.52	1.03E−07	−0.44	1.27E−05	0.85	2.54E−26
NFIL3	0.39	1.30E−04	−0.47	2.04E−06	−0.46	4.48E−06	0.85	4.49E−26
ITPRIP	0.29	4.77E−03	−0.27	1.11E−02	−0.27	1.01E−02	0.85	5.69E−26
PPP1R15B	0.24	2.06E−02	−0.31	3.14E−03	−0.29	5.05E−03	0.84	6.93E−26
SOCS3	0.28	6.45E−03	−0.27	8.37E−03	−0.30	4.31E−03	0.84	7.13E−26
SLC19A2	0.29	5.37E−03	−0.41	5.63E−05	−0.35	6.25E−04	0.84	1.44E−25
ADAMTS4	0.38	2.01E−04	−0.39	1.09E−04	−0.45	8.18E−06	0.84	1.61E−25
ZFP36	0.29	5.28E−03	−0.34	8.46E−04	−0.23	2.76E−02	0.84	2.86E−25
ADAMTS1	0.28	6.54E−03	−0.31	3.16E−03	−0.32	1.76E−03	0.83	4.31E−24
PHLDA1	0.38	2.37E−04	−0.47	2.38E−06	−0.46	5.78E−06	0.82	9.88E−24
SLC2A3	0.28	6.77E−03	−0.32	1.99E−03	−0.36	4.59E−04	0.82	1.25E−23
MT1M	0.24	2.38E−02	−0.34	9.14E−04	−0.26	1.47E−02	0.82	2.04E−23
MT1JP	0.26	1.31E−02	−0.39	1.38E−04	−0.34	9.10E−04	0.82	2.75E−23
MT1A	0.20	5.13E−02	−0.40	9.79E−05	−0.29	5.49E−03	0.82	3.22E−23
C11orf96	0.41	5.88E−05	−0.36	5.21E−04	−0.36	5.22E−04	0.82	6.40E−23
CDKN1A	0.33	1.65E−03	−0.36	3.92E−04	−0.32	1.74E−03	0.82	6.53E−23
CEBPD	0.27	9.00E−03	−0.32	1.72E−03	−0.28	6.34E−03	0.81	1.88E−22
APOLD1	0.28	6.42E−03	−0.23	2.85E−02	−0.26	1.37E−02	0.81	2.85E−22
SLCO4A1	0.32	2.26E−03	−0.45	7.30E−06	−0.40	1.00E−04	0.81	3.52E−22
RNF122	0.23	2.58E−02	−0.28	7.49E−03	−0.25	1.56E−02	0.81	4.16E−22
PELI1	0.25	1.89E−02	−0.25	1.49E−02	−0.32	1.87E−03	0.80	9.76E−22
SOCS2	0.26	1.15E−02	−0.24	2.09E−02	−0.32	1.89E−03	0.80	9.89E−22

### Identification of DEGs

Using the thresholds —log_2_ FoldChange— >0.5 and adjusted *p*-values <  0.05, we identified 1,347 upregulated and 1,023 downregulated genes in the GSE32537 dataset and 3,285 upregulated and 596 downregulated genes in the GSE24206 dataset. RRA was performed to integrate the DEGs identified in the GES32537 and GSE24206 datasets. Finally, 247 upregulated and 78 downregulated genes were identified (Table S2). The top 20 upregulated and the top 20 downregulated genes are shown in Fig. 4B.

### Construction of the PPI network and identification of hub genes

We uploaded 32 candidate hub genes into the STRING database to construct a PPI network (Fig. S2). Cytoscape software was used to visualize the interaction relationships among these hub nodes. The nodes with no connections were removed. The final network contained 20 nodes and 35 edges (Fig. 4C). The nodes with the top 5 degree of connectivity were interleukin-6 (*IL6*), MYC proto-oncogene (*MYC*), serpin family E member 1 (*SERPINE1*), thrombospondin-1 (*THBS1*), suppressor of cytokine signaling 3 (*SOCS3*), and CCAAT enhancer-binding protein delta (*CEBPD*).

The differentially expression genes *IL6*, *THBS1*, *SERPINE1,* and *SOCS3* were also included in the previously described nodes with great degree of connectivity in the PPI network (Fig. 4C). Therefore, we identified *IL6*, *SERPINE1*, *THBS1*, and *SOCS3* as hub genes, based on differential expression and connectivity.

### Validation of hub genes using additional GEO datasets

**Figure 5:**
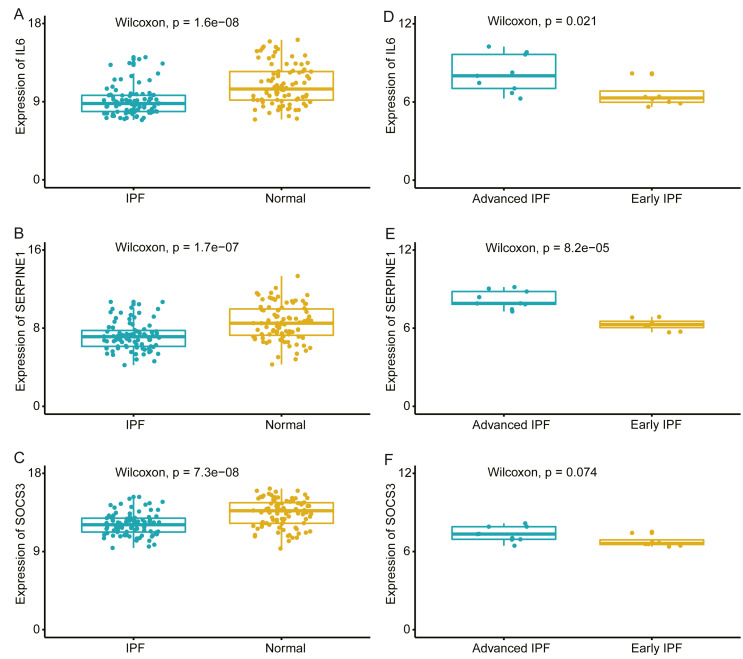
Validate the expression of hub genes in different groups. (A–C) In GSE47460 dataset, IL6, SERPINE1 and SOCS3 were significantly downregulated in IPF when compared with normal lung tissues. (D–E) In GSE24206 dataset, IL6 and SERPINE1 were significantly overexpressed in advanced IPF when compared with early IPF, (F) while the expression of SOCS3 was no statistical difference between the two groups (*p* = 0.074).

**Figure 6:**
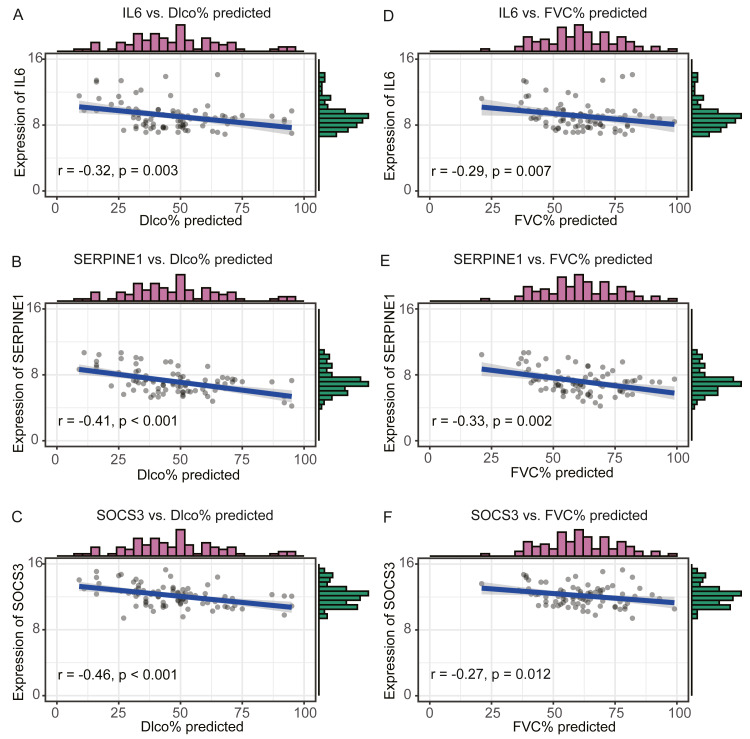
Validate the negative relationship between hub genes and lung function in GSE47460 dataset. (A–C) The relationship between expression levels of IL6, SERPINE1, SOCS3 and the Dlco% predicted of patients with IPF. Spearman correlation coefficients between Dlco% predicted and expression levels of IL6, SERPINE1, SOCS3 were −0.32 (*p* = 0.003), −0.41 (*p* < 0.001), and −0.46 (*p* < 0.001), respectively. (D–F) The relationship between expression levels of IL6, SERPINE1, SOCS3 and the FVC% predicted of patients with IPF. Spearman correlation coefficients between FVC% predicted and expression levels of IL6, SERPINE1, SOCS3 were −0.29 (*p* = 0.007), −0.33 (*p* = 0.002),and −0.27 (*p* = 0.012), respectively.

We compared the expression levels of hub genes between IPF and healthy lung tissues in the GSE47460 dataset (Figs. 5A–5C) and found that *IL6*, *SERPINE1,* and *SOCS3* were significantly downregulated in IPF patients compared with healthy controls. However, *THBS1* showed no significantly different expression (*p* = 0.75), which was excluded from further analyses (Fig. S3). In the GSE24206 dataset, the expression of the final three hub genes were lower in the early IPF group than in the advanced IPF group, although the expression of *SOCS3* did not differ significantly between the two groups (*p* = 0.074, Figs. 5D–5F). The Spearman correlation coefficients between Dlco% predicted and *IL6*, *SERPINE1, SOCS* were calculated as −0.32, −0.41, and −0.46, respectively (Figs. 6A–6C). The Spearman correlation coefficients between FVC% predicted and *IL6*, *SERPINE1*, *SOCS3* were calculated as −0.29, −0.33, and −0.27, respectively (Figs. 6D–6F).

## Discussion

In the present study, we found a significant inverse correlation between the red module and lung function by performing WGCNA on an IPF dataset. We identified three hub genes, including *IL6*, *SERPINE1,* and *SOCS3*. The expression levels of *IL6*, *SOCS3*, and *SERPINE1* were negatively correlated with lung function, and the advanced IPF patients had higher expression levels of these genes than early IPF patients in the validated datasets. The most important characteristic of patients with IPF is a decline in lung function, and declines of FVC and Dlco can predict mortality risk (Frankel & Schwarz, 2009; Nathan et al., 2011). However, the pathophysiologic mechanisms of lung dysfunction remain unclear. Our results may provide insights into the pathogenesis underlying the progression of lung function.

WGCNA is a bioinformatic algorithm and has been used to identify candidate biomarkers and therapeutic targets for many diseases, especially in cancer and neuroscience research (Giulietti et al., 2017; Li et al., 2020; Niemira et al., 2019; Rangaraju et al., 2018; Spiers et al., 2015; Zeleznik et al., 2020). We can identify clusters (modules) of highly correlated genes using WGCNA. WGCNA can systematically study the interconnectedness among all genes and convert gene expression data into a weighted co-expression network, which represents its most important advantage (Zhang & Horvath, 2005; Zhao et al., 2010). Based on the module significance, we can incorporate external clinical information into the network and identify key modules and hub genes, which are believed to play core roles in the pathogenesis of the disease.

The GSE32537 and GSE47460 datasets contain the transcriptomic profiles and clinical characteristics of the included subjects. Studies examining IPF have been previously performed using these datasets. Yang and colleagues (2013) analyzed the GSE32537 dataset and found that the high expression of cilium-associated genes was associated with increased microscopic honeycombing. McDonough and colleagues selected DEGs to construct co-expression networks by performing WGCNA on GSE47460 dataset and identified regulatory factors that were associated with co-expression networks in IPF (McDonough et al., 2019). In the present study, we selected the top 25% variant genes to perform WGCNA on patients with IPF in the GSE32537 dataset. We found that the red module had the strongest negative correlations with FVC% predicted and Dlco% predicted. We identified *IL6*, *SERPINE1,* and *SOCS3* as hub genes in the red module. We also validated the relationships between expression levels of these hub genes and lung function using independent datasets. To our knowledge, this study is the first to identify and validate genes that are negatively associated with lung function, based on transcriptomic files combined with the WGCNA approach.

In this study, the red module had the strongest negative correlation with lung function. The enrichment analysis of genes in the red module showed that they were primarily associated with inflammatory and immune responses, which indicated that the inflammatory and immune pathways are involved in the pathophysiologic mechanisms of lung dysfunction. Although multicenter trials of anti-inflammatory drugs for IPF treatment have failed (Farrand et al., 2020; King et al., 2009; Raghu et al., 2004; Raghu et al., 2017; Raghu et al., 2008), the immune system continues to be regarded as playing an important role in the development of fibrosis (Heukels et al., 2019; Wynn, 2011). Furthermore, many studies have also confirmed that changes in immune activity or the proportions of immune cell populations may be associated with declines in lung function (Adegunsoye et al., 2016; Gilani et al., 2010; Xue et al., 2013).

Interestingly, all three hub genes were downregulated in the IPF group compared with their levels in the healthy group. We speculated that hub genes had different molecular functions under different conditions. IL6 is a multifunctional cytokine, belonging to the IL-6 family of cytokines. Various cells, including alveolar macrophages, lung fibroblasts, and fibrocytes, can express and secret IL6 (Shahar et al., 1996). Takizawa and colleagues observed that IL6 concentrations were significantly higher in bronchoalveolar lung fluid (BALF) from IPF patients than in BALF from healthy controls (Takizawa et al., 1997). However, in this study, when compared with healthy lung tissues, the expression of *IL6* was lower in IPF tissues. A previous study demonstrated that IL6 promoted the proliferation of IPF lung fibroblasts but inhibited the proliferation of normal lung fibroblasts (Moodley et al., 2003b). In another study, in lung fibroblasts derived from IPF patients, IL6 contributed to resistance against Fas-induced apoptosis by increasing the expression of the anti-apoptotic protein BCL-2, whereas normal lung fibroblasts became more sensitive to Fas-induced apoptosis, which was mediated by the increased expression of the pro-apoptotic protein Bax when exposed to IL6 (Moodley et al., 2003a). In the present study, the expression of *IL6* was negatively associated with lung function, which also indicated IL6 promoted the progression of IPF.

Suppressor of cytokine signaling-3 (SOCS3) is a well-known regulatory cornerstone of intracellular signaling. SOCS3 not only acts as a feedback inhibitor of the JAK/STAT signaling pathway but can also regulate many cytokines, growth factors, and hormones associated with many cellular processes (Mahony et al., 2016). Whether SOCS3 acts to protect against or promote disease progression depends on the cells and pathological processes in which it is expressed, especially in innate and adaptive immunity (Kubo, Hanada & Yoshimura, 2003; Yasukawa et al., 2003). A previous study demonstrated that silencing *Socs3* in a rat diastolic heart failure model was able to significantly diminish myocardial fibrosis and the inflammatory response (Gao et al., 2019). SOCS3 function has also been studied in lung diseases (Gao & Ward, 2007). Studies have revealed that SOCS3 acts as a pro-inflammatory molecule, by suppressing the IL-6-gp130 signaling pathway, and mice lacking *Socs3* in macrophages and neutrophils were resistant to LPS-induced shock (Yasukawa et al., 2003). Aboulhoda studied age-dependent *SOCS3* expression and myocardial fibrosis, and found that *SOCS3* activity was correlated with myocardial fibrosis (Aboulhoda, 2017). IPF is an aging-related disease, but the role played by *SOCS3* in pulmonary fibrosis has not been well-studied. The present study revealed a negative correlation between *SOCS3* expression and lung function in IPF patients, but the detailed mechanisms require further study.

Serpin Family E Member 1 (SERPINE1), also known as plasminogen activator inhibitor-1 (PAI-1), is the primary inhibitor of plasminogen activators, such as tissue-type plasminogen activator (t-PA) and urokinase-type plasminogen activator (u-PA), and acts as a major regulator of the fibrinolytic system. Impaired fibrinolytic activity is a common characteristic of acute and chronic inflammatory lung diseases, especially pulmonary fibrosis (Marudamuthu et al., 2015). Eitzman studied transgenic mice that either overexpressed or were completely deficient in murine *Serpine1*, and found that higher levels of *Serpine1* expression can increase collagen accumulation following inflammatory lung injury (Eitzman et al., 1996). Osterholzer and colleagues studied type-II alveoli epithelial cells in a lung injury model and found results consistent with those reported by previous studies (Osterholzer et al., 2012). Senoo and colleagues directly suppressed the expression of *Serpine1* in mice, through the intrapulmonary administration of *Serpine1*-siRNA, to reduce pulmonary fibrosis. They found that the suppression of epithelial-to-mesenchymal transformation may be involved in IPF (Senoo et al., 2010). The present study confirmed a negative correlation between the expression level of *SERPINE1* and IPF.

We identified *IL6*, *SOCS3*, and *SERPINE1* as IPF hub genes that were negatively associated with lung function. These hub genes may serve as therapeutic targets for IPF treatment. The downregulation of *SERPINE1* has been shown to attenuate pulmonary fibrosis (Senoo et al., 2010), indicating the reliability of the our results. However, the present study also has some limitations. First, the study is based on bioinformatics analysis, and the results remain to be verified by further research. Second, datasets that met the inclusion criteria were rare, which may decrease the statistical effectiveness. Finally, the difference of GeneChips between the datasets may also affect the reliability of the results.

## Conclusions

In summary, we performed WGCNA on an IPF dataset. Among 14 modules, the red module was identified as a key module because it displayed the strongest correlation with lung function. Genes in the red module were primarily enriched in inflammatory and immune pathways. *IL6*, *SOCS3,* and *SERPINE1* were identified as hub genes from the red module. We also found that *IL6*, *SOCS3,* and *SERPINE1* were negatively associated with lung function in IPF patients. These results may suggest that further study is warranted to investigate the roles played by hub genes in IPF progression. Based on this research, the proteins encoded by these hub genes may serve as biomarkers for IPF severity and may represent therapeutic targets for IPF.

##  Supplemental Information

10.7717/peerj.9848/supp-1 Supplemental Information 1 Detect and remove the outliers When the threshold was set as 60, the GSM806234, GSM806335 were outliers and were removed.

10.7717/peerj.9848/supp-2 Supplemental Information 2 Construct PPI network using STRING database MT1JP, one of the 32 candidate hub genes, is a pseudogene and dose not code protein, so there were 31 nodes in PPI network. Colored nodes are the first shell of interactors, while white nodes represent second shell of interactors. Empty nodes represent proteins of unknown 3D structure, while filled nodes indicate that some 3D structure is known or predicted.

10.7717/peerj.9848/supp-3 Supplemental Information 3 Expression of THBS1 The expression of THBS1 in IPF group compared with healthy group.

10.7717/peerj.9848/supp-4 Supplemental Information 4 Summary of those gene expression datasets used in this study

10.7717/peerj.9848/supp-5 Supplemental Information 5 The result of RRA analysis

10.7717/peerj.9848/supp-6 Supplemental Information 6 Data for WGCNA

10.7717/peerj.9848/supp-7 Supplemental Information 7 Result of WGCNA

## Additional Information and Declarations

### Data Availability

The following information was supplied regarding data availability: Data are available at NCBI GEO: GSE32537, GSE24206, GSE47460 and the raw data and results of WGCNA are available in the Supplementary Files.
