# Supplementary figures and images for: Identification of key modules and hub genes associated with lung function in idiopathic pulmonary fibrosis
Source: PeerJ. 2020 Sep 8;8:e9848. doi: 10.7717/peerj.9848 (PMC7485506; doi:10.7717/peerj.9848)

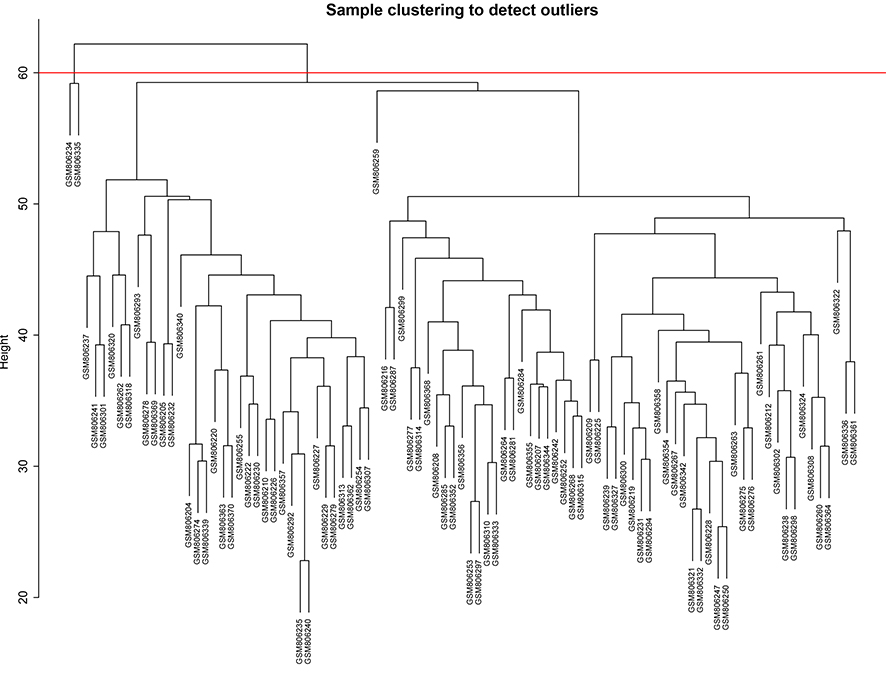

Supplement: Supplemental Information 1 — When the threshold was set as 60, the GSM806234, GSM806335 were outliers and were removed. [file peerj-08-9848-s001.jpg]

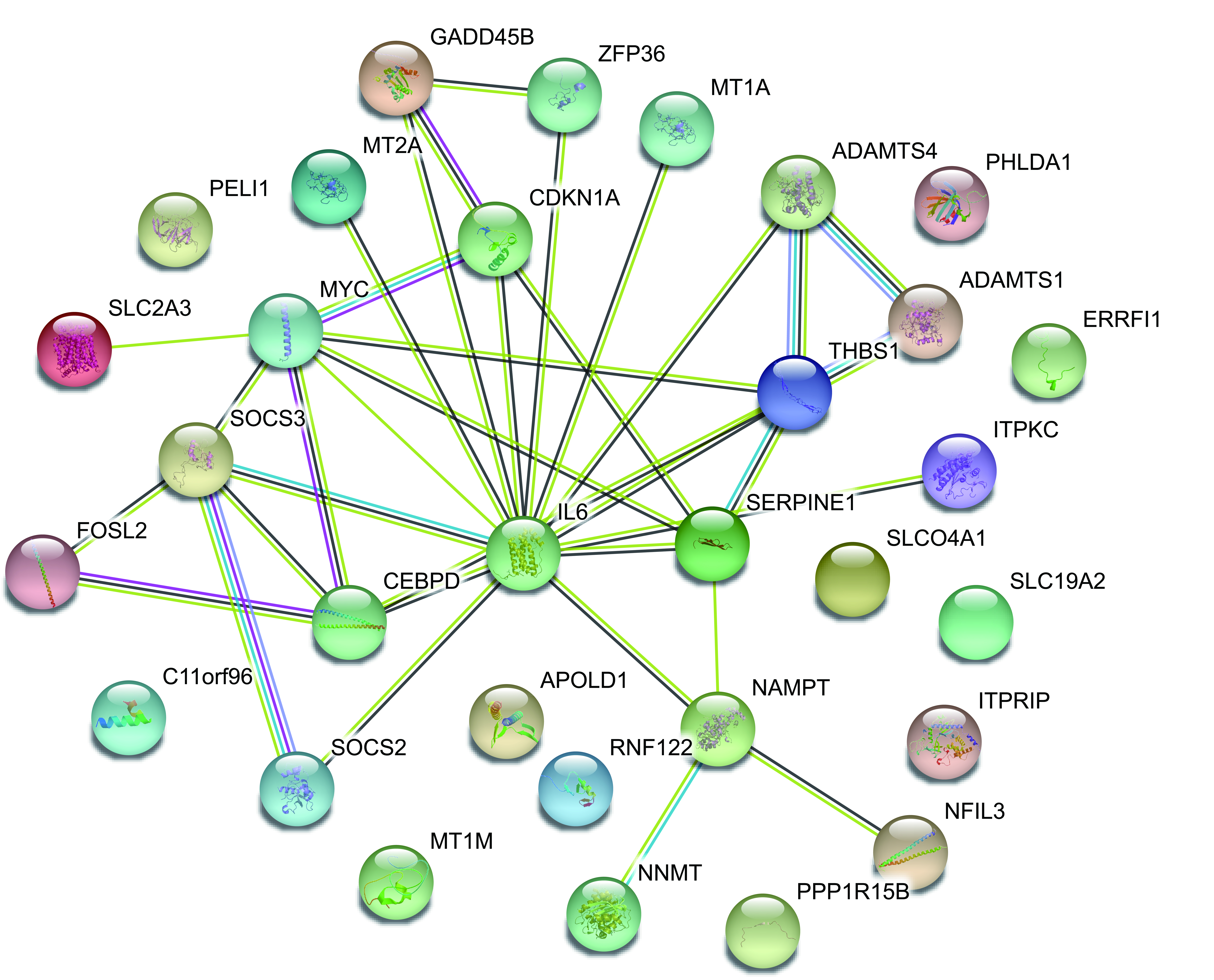

Supplement: Supplemental Information 2 — MT1JP, one of the 32 candidate hub genes, is a pseudogene and dose not code protein, so there were 31 nodes in PPI network. Colored nodes are the first shell of interactors, while white nodes represent second shell of interactors. Empty nodes represent proteins of unknown 3D structure, while filled nodes indicate that some 3D structure is known or predicted. [file peerj-08-9848-s002.jpg]

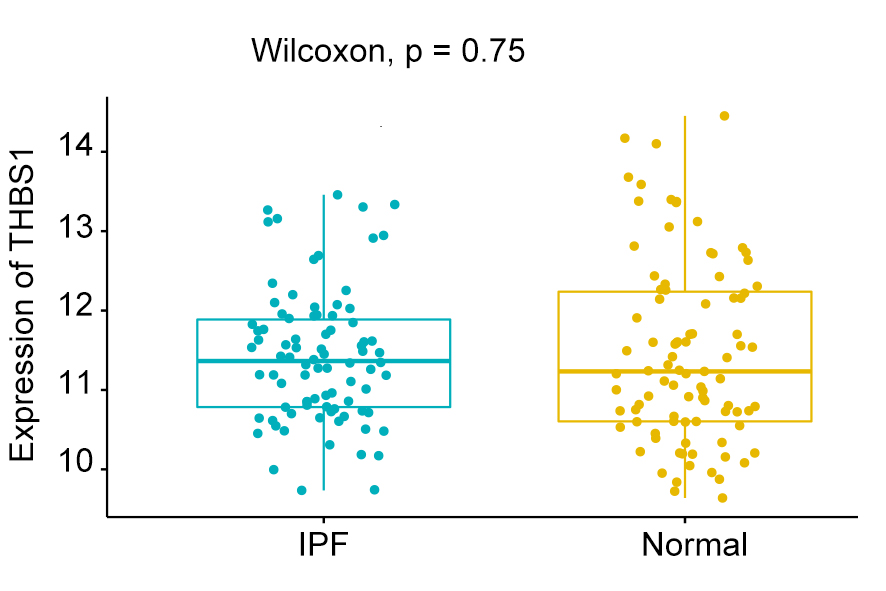

Supplement: Supplemental Information 3 — The expression of THBS1 in IPF group compared with healthy group. [file peerj-08-9848-s003.jpg]
