# Supplementary material for: Identification of key modules and hub genes associated with lung function in idiopathic pulmonary fibrosis
Source: PeerJ. 2020 Sep 8;8:e9848. doi: 10.7717/peerj.9848 (PMC7485506; doi:10.7717/peerj.9848)
Supplement: Supplemental Information 4 [file peerj-08-9848-s004.docx]

| GSE number | Samples | Source type | Year | Platform | Auther |
| --- | --- | --- | --- | --- | --- |
| GSE32537 | 93 IPF and 50 healthy | Lung tissues | 2013 | GPL6244 | David Schwartz |
| GSE24206 | 8 early IPF, 9 advanced IPF and 6 healthy | Lung tissues | 2011 | GPL570 | Eric B. Meltzer |
| GSE47460 | 86 IPF and 91 healthy | Lung tissues | 2013 | GPL6480 | John Tedrow |
